# MoS_2_ quantum dot-decorated CNT networks as a sulfur host for enhanced electrochemical kinetics in advanced lithium–sulfur batteries†

**DOI:** 10.1039/d4na00068d

**Published:** 2024-10-22

**Authors:** Meng Wei, Hanqing Lu, Zhen Wang, Baowen Lu, Pengtao Wang, Xinxin Zhang, Bingjie Feng, Yingjie Xie, Tao Zhang, Guanghui Liu, Song Xu

**Affiliations:** a School of Materials Science and Engineering, Zhengzhou University of Aeronautics Zhengzhou 450046 China weimeng1005@zua.edu.cn songxu@zua.edu.cn; b Collaborative Innovation Center of Aviation Economy Development Henan Province China

## Abstract

The slow redox kinetics and shuttle effect of polysulfides severely obstruct the further development of lithium–sulfur (Li–S) batteries. Constructing sulfur host materials with high conductivity and catalytic capability is well acknowledged as an effective strategy for promoting polysulfide conversion. Herein, a well-designed MoS_2_ QDs-CNTs/S@Ni(OH)_2_ (labeled as MoS_2_ QDs-CNTs/S@NH) cathode was synthesized *via* a hydrothermal process, in which conductive polar MoS_2_ quantum dot-decorated carbon nanotube (CNT) networks coated with an ultrathin Ni(OH)_2_ layer acted as an efficient electrocatalyst. MoS_2_ QD nanoparticles with a high conductivity and catalytic nature can enhance the kinetics of polysulfide conversion, expedite Li_2_S nucleation, and decrease the reaction energy barrier. The thin outer Ni(OH)_2_ layer physically confines active sulfur and meanwhile provides abundant sites for adsorption and conversion of polysulfides. Benefiting from these merits, a battery using MoS_2_ QDs-CNTs/S@NH as the sulfur host cathode exhibits excellent electrochemical performances with rate capabilities of 953.7 mA h g^−1^ at 0.1C and 606.6 mA h g^−1^ at 2.0C. A prominent cycling stability of a 0.052% decay rate per cycle after 800 cycles is achieved even at 2C.

## Abstract 2

Schematic illustration of the synthesis process of MoS_2_ QDs-CNTs/S@NH composites.

## Graphical abstract

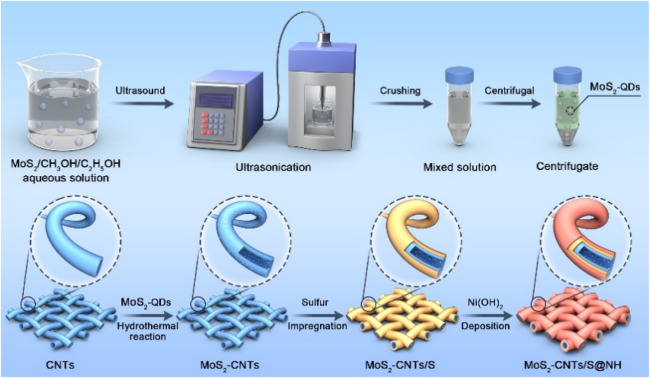

## 1 Introduction

Rapid global energy growth and a low-carbon economy have driven the vigorous development of higher energy-density devices.^1,2^ Almost every portable device, such as smart grids, portable electronics, and electric vehicles, that use electricity has benefited from the development of rechargeable lithium-ion batteries (LIBs).^3,4^ However, the most advanced commercial lithium-ion battery nearly reaches its theoretical limitation owing to the “Li^+^ intercalation” mechanism, which restricts its developing potential in competitive future application.^5,6^ In order to accomplish the goal of high energy density over 500 W h kg^−1^, it is an urgent need to explore new battery systems.^7,8^ In this case, lithium–sulfur (Li–S) batteries with a high theoretical energy density of 2600 W h kg^−1^, environmentally friendly nature and abundant reserves of sulfur have been regarded as the promising candidate for next generation batteries.^9,10^

However, the application of Li–S batteries is still challenging. There are several technical problems that have not been solved, such as sluggish reaction kinetics, dielectric sulfur and its final product Li_2_S, soluble lithium polysulfides (LiPSs), and sulfur volume expansion.^11,12^ The most serious one is the dissolution of polysulfides and their shuttling effect (the so-called shuttle effect) caused by soluble lithium polysulfides (LiPSs), which can shuttle between the cathode and Li anode, resulting in the loss of active materials and low utilization of active sulfur.^13,14^ The dissolution of long-chain LiPSs not only increases the viscosity of the electrolyte, but also increases charge transfer resistance.^15^ Moreover, with the soluble LiPSs forming the nucleation barrier of solid Li_2_S_2_/Li_2_S, parts of them are lost from the electrode, leading to incapable transformation from liquid high-order lithium polysulfides (Li_2_S_*n*_, 4 ≤ *n* ≤ 8) to solid lithium polysulfides (Li_2_S_*n*_, 1 ≤ *n* ≤ 2) under high current density.^16,17^ Meanwhile, the converse transition of solid Li_2_S to dissolved LiPSs needs to overcome additional activation energy due to the aggregation of Li_2_S produced. Solid–liquid–solid phase transitions and their sluggish kinetic conversion problems sharply reduce the capacity, cycling life, and coulombic efficiency of Li–S batteries.^18,19^

Traditional physical confinement and chemisorption have been proven effective in dealing with the shuttle effect; however, they only alleviate the polysulfide shuttling to some extent and cannot solve the problem fundamentally.^20,21^ The root cause of the shuttle effect is the slow transformation of the liquid phase intermediate lithium polysulfide (Li_2_S_*n*_, 4 ≤ *n* ≤ 8) to solid product Li_2_S_2_/Li_2_S, which continuously accumulates in the cathode and diffuses to the lithium anode driven by the concentration gradient and electric field.^22,23^ The electrochemical reaction kinetics can likely slow down inside the battery. Hence, a new perspective of electrocatalytic reaction kinetics that accelerates the sulfur “solid–liquid–solid” conversion process will be required.^24–27^

Enormous endeavors have been made to overcome this issue. Conductive materials (*e.g.*, carbon-based materials,^28–31^ metal oxides,^32,33^ conductive polymers^34^) have been widely used as the host materials to construct a conductive network and weaken the dissolution of polysulfide. However, the low-polar carbon materials are limited to weak interactions with LiPSs and can only trap LiPSs by physical confinement and chemical adsorption, which cannot effectively suppress the shuttle effect and promote the conversion kinetics of the battery.^35,36^ In this case, polar metal compounds with strong adsorption capacity for LiPSs have been developed successively to promote ion adsorption and migration efficiently, such as VS_2_,^37^ TiS_2_,^38^ WS_2_,^39^ ReS_2_,^40^ TiO_2_,^41^ MnO_2_,^42^ VO_2_,^43^ VN,^44,45^ and so on. Among these, molybdenum disulfide (MoS_2_), with the change of lattice plane, high surface area and plentiful active sites, facilitates the strengthening of the overall absorption of Mo ions with negatively charged polysulfides.^46^ Meanwhile, MoS_2_ is a typical two-dimensional transition metal sulfide that forms zero-dimensional MoS_2_ quantum dots (MoS_2_ QDs) when the MoS_2_ QDs size is reduced to 10 nm and below. The advantages such as unsaturated bonds, strong binding energy, and abundant polar active sites are expected to greatly enhance the kinetics of polysulfides redox reactions.^47^

Herein, a novel architecture of MoS_2_ QDs-CNTs/S@NH was constructed to accommodate sulfur, followed by ultrathin Ni(OH)_2_ layer encapsulation. The MoS_2_ QDs-CNTs/S@NH cathode was prepared by a hydrothermal process, in which nanosized MoS_2_ QDs anchored on CNTs networks were used in both the sulfur host and multifunctional electrocatalysts to catalyze the LiPSs conversion. Benefiting from the synergy between conductive MoS_2_ QDs-CNTs framework and ultrathin Ni(OH)_2_ layer coating, the shuttle effect was effectively suppressed, and catalytic conversion of polysulfides showed a significant improvement. The MoS_2_ QDs-CNTs/S@NH-fabricated batteries with a high sulfur content of 70 wt% show excellent performance both in long cycling stability and rate capability. The MoS_2_ QDs-CNTs/S@NH cathode offers enhanced performance with an excellent capacity retention of 59.2% over 800 cycles at 2C. The improved electrochemical performance can be attributed to the design of the cathode, whose CNTs network provides fast electron/ion transfer and polar MoS_2_ QDs offering lots of active sites to adsorb polysulfides through chemical interactions. The thin Ni(OH)_2_ layer coats as a physically protective shield, not only confining active sulfur but also effectively inhibiting polysulfides shuttle through chemical interactions.

## 2 Experimental section

### 2.1 Materials

Molybdenum disulfide (Analytical pure, Shanghai Aladdin Reagent Co., Ltd.); nickel sulfate hexahydrate (AR, 99.9%), ammonium persulfate (AR, 98%) (all analytically pure, Shanghai Aladdin Biochemical Technology Co., Ltd.), ammonia solution (Analytically pure, Xi long Chemical Co., Ltd.), carbon nanotube multi-walled carbon nanotubes (ID: 5–10 nm, OD: 20–30 nm, length: 10–30 nm), sublimed sulfur, *N*-methyl pyrrolidone (NMP) (all analytically pure, Sinopharm Group Chemical Reagents Co., Ltd.); lithium sulfide, thioacetamide 1,3-dioxane (DOL), ethylene glycol dimethyl ether (DME) (Analytically pure, Shanghai Aladdin Reagent Co., Ltd.); LA133 Water system binder (Guangdong Candle New Energy Technology Co., Ltd.).

### 2.2 Preparation of MoS_2_ QDs

Typically, methanol aqueous solution (40 vol%) and ethanol aqueous solution (45 vol%) were mixed in a volume ratio of 1 : 1. 100 mg of molybdenum disulfide powder was dispersed in 40 mL methanol/ethanol solution and ultrasonically treated at room temperature for 2 h. Then, the mixture was dispersed in an ultrasonic cell crusher for 2 h with low-temperature control. After centrifugation at 11 000 rpm for 10 min 3 times, the MoS_2_ QDs were obtained and stored at 4 °C.

### 2.3 Preparation of MoS_2_ QDs-CNTs

0.15 g of hydroxylated carbon nanotubes were dispersed in 50 mL distilled water and sonicated for 30 min. Subsequently, the molybdenum disulfide quantum dot (MoS_2_ QDs) solution was uniformly mixed into the CNTs dispersion and stirred for 45 min (mass ratio of 50 : 1 for CNTs to MoS_2_ QDs). The mixture was poured into a 100 mL hydrothermal reactor and reacted for 12 h at 180 °C. After cooling to room temperature, the resulting material is extracted and filtered, then washed several times with deionized water and ethanol to remove surface impurities. Finally, the experimental material was freeze-dried for 24 h to obtain a pure MoS_2_ QDs-CNTs sample.

### 2.4 Preparation of MoS_2_ QDs-CNTs/S

MoS_2_ QDs-CNTs/S composite material was prepared using a simple melt diffusion method. MoS_2_ QDs-CNTs and sublimed sulfur were mixed in a mass ratio of 3 : 7, and the mixture was ground for 40 min. Subsequently, the mixture was transferred to a stainless steel reactor and heated at 155 °C in an argon atmosphere for 12 h. After cooling to room temperature, the MoS_2_ QDs-CNTs/S composite material was obtained.

### 2.5 Preparation of MoS_2_ QDs-CNTs/S@NH

The Ni(OH)_2_ layer was coated on the surface of the MoS_2_ QDs-CNTs/S precursor using a surface chemical precipitation method. The mixed solution was formed by dispersing 4 mL nickel sulfate hexahydrate and 2.5 mL ammonium persulfate in 100 mL deionized water. Subsequently, 0.45 g MoS_2_ QDs-CNTs/S composite was dissolved in the mixed solution to form a stable solution. Then, 10 mL of concentrated ammonia solution was added, followed by stirring at room temperature for 30 min and standing for 20 min. The product was centrifuged at 5000 rpm for 10 min and washed several times. Finally, the MoS_2_ QDs-CNTs/S@NH material was obtained after drying in a vacuum oven at 60 °C for 12 h.

### 2.6 Preparation of the MoS_2_ QDs-CNTs/S@NH cathode

The MoS_2_ QDs-CNTs/S@NH composite with conductive carbon black was mixed uniformly in a mass ratio of 8 : 1. Subsequently, the powder was added to the LA133 solution in a weight ratio of 9 : 1 and stirred for 12 h to prepare a homogeneous slurry. The prepared slurry was coated onto an aluminum foil with a thickness of 300 μm and subsequently dried in a vacuum drying oven for 12 h. After drying, the MoS_2_ QDs-CNTs/S@NH cathode was cut into disks with a diameter of 12 mm for assembly into CR2032 coin cells.

### 2.7 Material characterization

The crystallinity and structure of the samples were characterized using X-ray diffraction (XRD) with a Bruker D2 PHASER instrument. Scanning electron microscopy (SEM) utilizing the ZEISS Gemini 300 and transmission electron microscopy (TEM) employing the JEOL JEM-2100Plus were employed to characterize the morphology and structure of the samples. X-ray photoelectron spectroscopy (XPS) measurements were conducted to analyze the chemical states of elements and material composition. The sulfur content in the MoS_2_ QDs-CNTs/S@NH composite was characterized using a thermogravimetric analyzer (TG, TG 209F3). The concentration of Li_2_S_6_ in the solution was characterized using a UV-visible spectrophotometer (UV-vis, UV-5500PC), facilitating the analysis of lithium polysulfide adsorption properties in the samples.

### 2.8 Electrochemical measurements

The MoS_2_ QDs-CNTs/S@NH composite, conductive superphosphate and binder LA133 were mixed in a ratio of 7 : 2 : 1 to form a uniform slurry. The slurry was cast onto an aluminum foil, vacuum-dried at 60 °C for 12 h and subsequently punched into discs with a diameter of 12 mm. The coin cells were assembled with MoS_2_ QDs-CNTs/S@NH as the cathode, Celgard 2500 as the separator, lithium foil as the anode and 1 M LiTFSI solution in a mixed solvent of 1,3-dioxolane (DOL) and 1,2-dimethoxyethane (DME) in a volume ratio of 1 : 1 in a glove box filled with Ar (H_2_O, O_2_ < 0.1 ppm). The sulfur content of the whole cathode is approximately 70 wt%. The sulfur mass loading on the cathode ranges between 2 and 2.7 mg cm^−2^. Furthermore, the ratio of the cathode electrolyte to sulfur is 13 μL mg^−1^. The electrochemical performance of the system was characterized using the LAND testing system and CHI-604E electrochemical workstation. Cyclic voltammetry (CV) was tested at a scan rate of 0.1 mV s^−1^, and electrochemical impedance spectroscopy (EIS) was performanced by a frequency range of 100 kHz to 0.01 Hz.

## 3 Results and discussion

**Fig. 1:**
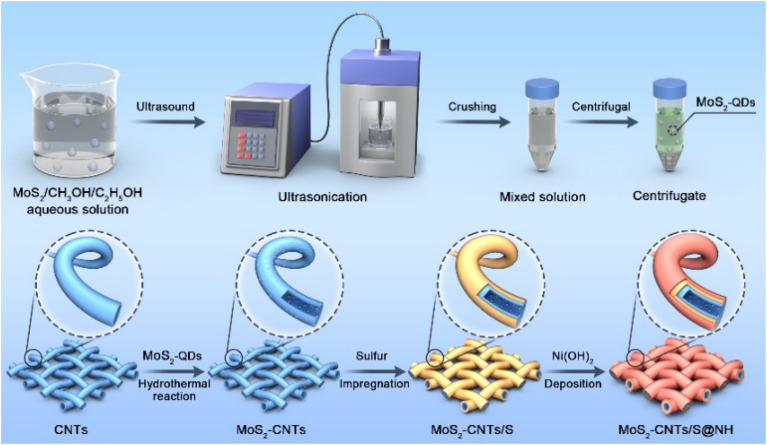
Schematic of the synthesis process of MoS_2_ QDs-CNTs/S@NH composites.

The synthetic process of the MoS_2_ QDs-CNTs/S@NH material is illustrated in Fig. 1. The MoS_2_ QDs-CNTs/S@NH shows an effective design with CNTs constructed as a conductor network and MoS_2_ QDs homogeneously and tightly anchored on the CNTs networks. The active sulfur is loaded on the surface of the CNTs network, and a thin Ni(OH)_2_ layer is coated outside as a protective shield.

**Fig. 2:**
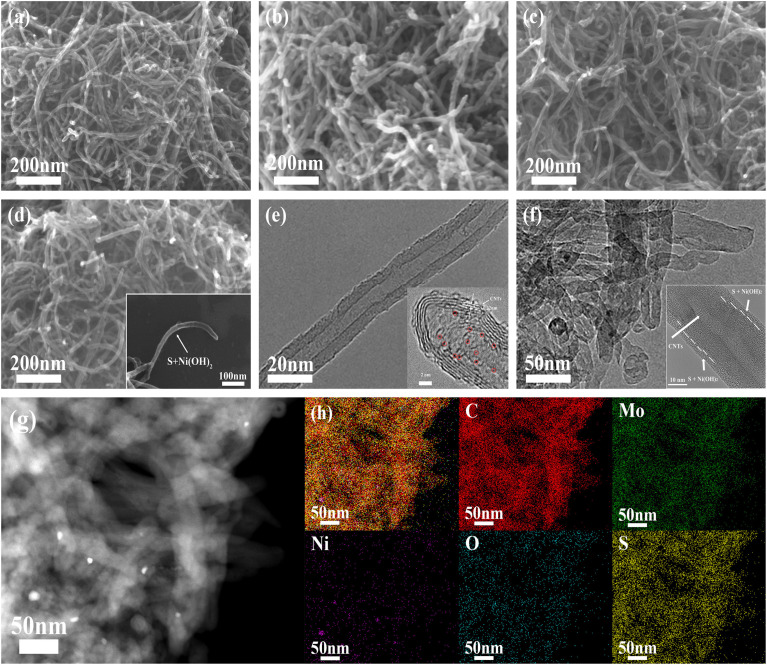
SEM images of (a) CNTs, (b) MoS_2_ QDs-CNTs, (c) MoS_2_ QDs-CNTs/S, and (d) MoS_2_ QDs-CNTs/S@NH. (e) HRTEM image of MoS_2_ QDs-CNTs. (f and g) High-magnification TEM image of MoS_2_ QDs-CNTs/S@NH and the EDS mapping.

Fig. 2(a)–(d) illustrates the morphological evolution process of the CNTs powder, MoS_2_ QDs-CNTs, MoS_2_ QDs-CNTs/S and MoS_2_ QDs-CNTs/S@NH, respectively. As shown in Fig. 2(a), the surface of naked carbon nanotubes (CNTs) is extremely smooth, with an outer diameter of approximately 30–40 nm+. Through the hydrothermal reaction process, MoS_2_ quantum dots (QDs) were successfully decorated onto the surface of carbon nanotubes, forming the MoS_2_ QDs-CNTs composite shown in Fig. 2(b). Compared to the bare carbon nanotubes, the MoS_2_ QDs-CNTs sample exhibits a relatively rough surface while preserving its initial one-dimensional structure. The MoS_2_ QDs-CNTs/S sample was prepared *via* the sulfur impregnation method, as shown in Fig. 2(c). The surface of MoS_2_ QDs-CNTs/S becomes slightly smoother than MoS_2_ QDs-CNTs, which can be attributed to the fact that MoS_2_ quantum dots (QDs) on the surface have been covered after sulfur loading. To further suppress the shuttle effect of polysulfides, the MoS_2_ QDs-CNTs/S composite was subsequently coated with a thin Ni(OH)_2_ layer. From Fig. 2(d) and the inset, a polar layer of Ni(OH)_2_ is clearly coated onto the surface of the MoS_2_ QDs-CNTs/S sample, achieving the successful construction of a core–shell structured MoS_2_ QDs-CNTs/S@NH cathode. Fig. 2(e) shows the high-resolution transmission electron microscope (HRTEM) image of MoS_2_ QDs-CNTs in which small MoS_2_ QDs (<5 nm) are evenly decorated on the surface of carbon nanotubes and some are encapsulated in narrow channels. The HRTEM is further proof of the coexistence of MoS_2_ QDs and CNTs. HRTEM images (Fig. 2(e)) show highly parallel and ordered lattice fringes, demonstrating the MoS_2_ QDs are well crystallized. The *d*-spacing of MoS_2_ QDs is 0.25 nm due to the (103) faces of MoS_2_ crystals. From the TEM image in Fig. 2(f) and the insertion, it is clear that the MoS_2_ QDs-CNTs nucleus is surrounded by a thin Ni(OH)_2_ shell. The high-magnification TEM image (Fig. 2(f)) confirms the nanosized MoS_2_ QDs decorated on the network structure of carbon nanotubes and coated by a thin Ni(OH)_2_ layer, which coincides with the SEM image in Fig. 2(d).

The as-prepared MoS_2_ QDs-CNTs/S and its energy dispersive X-ray (EDX) analysis are also investigated (Fig. S1†). After coating with Ni(OH)_2_, the MoS_2_ QDs-CNTs/S@NH and corresponding EDS mappings are shown in Fig. 2(g). Fig. 2(g) depicts the C, Ni Mo, S and O elements detected in the MoS_2_ QDs-CNTs/S@NH composite material, which demonstrates a homogeneous distribution of elements (C, O, Mo, S) in composites and the presence of the Ni(OH)_2_ layer. The additional Ni(OH)_2_ layer not only prevents the migration of sulfur but also facilitates the polar chemical adsorption to immobilize the dissolved polysulfides.

**Fig. 3:**
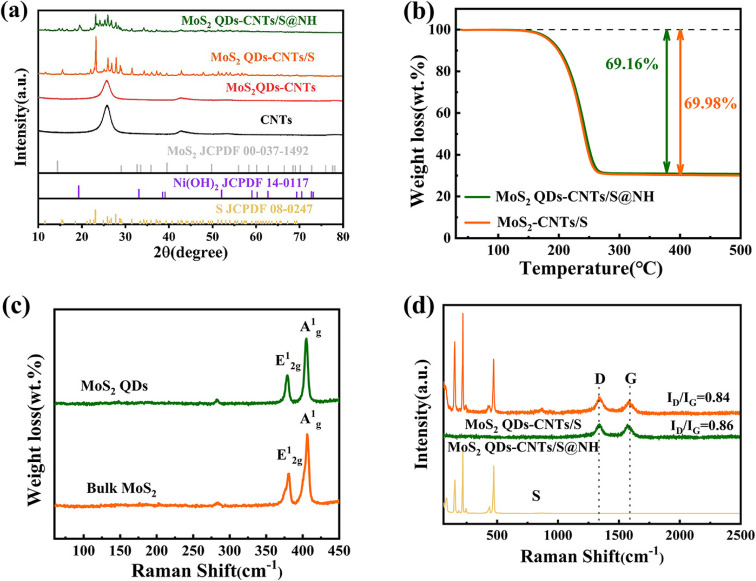
(a) XRD patterns of S, CNTs, MoS_2_ QDs-CNTs, Ni(OH)_2_, MoS_2_ QDs-CNTs/S and MoS_2_ QDs-CNTs/S@NH composite materials; (b) TG curves of MoS_2_ QDs-CNTs/S and MoS_2_ QDs-CNTs/S@NH; (c) Raman spectra of MoS_2_ QDs and bulk MoS_2_. (d) Raman spectra of MoS_2_ QDs-CNTs/S and MoS_2_ QDs-CNTs/S@NH.

X-ray diffraction (XRD) was performed to evaluate the purity and crystalline phases of the sample. To ensure the successful synthesis of MoS_2_ QDs-CNTs/S@NH, pure MoS_2_ QDs and Ni(OH)_2_ were both prepared and tested. As shown in Fig. 3(a), the diffraction peaks at 19.25°, 33.06° and 38.54° correspond to the (001), (100) and (101) planes of Ni(OH)_2_, confirming the successful preparation of nickel hydroxide.^48^ However, clear peaks related to molybdenum disulfide (MoS_2_) were not detected in the XRD spectrum of MoS_2_ QDs-CNTs. This can be attributed to the low mass ratio of MoS_2_ QDs as well as the incorporation of some MoS_2_ QDs into the lattice of carbon nanotubes without altering the crystal structure. The strong diffraction peaks of sublimed sulfur are detected in MoS_2_ QDs-CNTs/S and MoS_2_ QDs-CNTs/S@NH composites, indicating the presence of a well-defined S_8_ crystal structure (S JCPDS 08-0247). While the Ni(OH)_2_ was coated, it was noted that no distinct diffraction peaks of Ni(OH)_2_ were observed in the MoS_2_ QDs-CNTs/S@NH sample, which could be attributed to either a low content of Ni(OH)_2_ or the overlapping of weak Ni(OH)_2_ peaks with the strong diffraction peaks of sulfur. The intensity of the sulfur peaks was relatively weaker in MoS_2_ QDs-CNTs/S@NH due to the Ni(OH)_2_ layer, which agrees with the Raman analysis. The sulfur content of the MoS_2_ QDs-CNTs/S and MoS_2_ QDs-CNTs/S@NH samples was confirmed by thermogravimetric analysis (TGA). As shown in Fig. 3(b), a weight loss of 69.98% for the MoS_2_-CNTs/S sample can be observed when the temperature increases to 300 °C, which can be attributed to the evaporation of sulfur. For MoS_2_ QDs-CNTs/S@NH, approximately 69.16% sulfur loss was observed, slightly lower than that of MoS_2_ QDs-CNTs/S. This slight difference can be attributed to the decomposition of Ni(OH)_2_. Such low content of Ni(OH)_2_ is in accord with the fact that the diffraction peak of Ni(OH)_2_ was undetectable in the XRD analysis.

Raman spectroscopy was conducted to study the microstructure of bulk MoS_2_, MoS_2_ QDs, MoS_2_ QDs-CNTs/S and MoS_2_ QDs-CNTs/S@NH. In Fig. 3(c), the characteristic peaks A_g_^1^ and E_2g_^1^ of MoS_2_ QDs were perfectly consistent with the peaks of bulk MoS_2_, which are located at 406.7 cm^−1^ and 381 cm^−1^.^49,50^ The Raman spectrum of MoS_2_ QDs-CNTs/S and MoS_2_ QDs-CNTs/S@NH with two main modes, the D and the G band were located at 1337 cm^−1^ and 1573 cm^−1^, which reveals the disordered vibration of carbon atoms with defects and two-dimensional vibration in the hexagonal lattice of sp^2^-bonded carbon atoms (Fig. 3(d)). In general, the relative intensity ratio *I*_D_/*I*_G_ is an indication of the carbon nanotube quality. The *I*_D_/*I*_G_ value of MoS_2_ QDs-CNTs/S@NH (*I*_D_/*I*_G_ 0.86) is smaller than that of MoS_2_ QDs-CNTs/S (*I*_D_/*I*_G_ 0.84), indicating that combine with Ni(OH)_2_ does not affect the defects material after the sonication or hydrothermal process. The characteristic Raman peak of sulfur was observed on MoS_2_ QDs-CNTs/S, and three sharp peaks of the S/CNT/G film below 600 cm^−1^ could be assigned to the S–S bond in the composites. It is noteworthy that the Raman spectra of MoS_2_ QDs-CNTs/S@NH showed no sulfur peak, indicating that S_8_ molecules were well fixed in the Ni(OH)_2_ thin layer without significant molecular vibrations. This result further proves that the structure of Ni(OH)_2_ on the surface of sulfur can well limit the diffusion of sulfur, thereby improving the utilization rate and stability of sulfur.

**Fig. 4:**
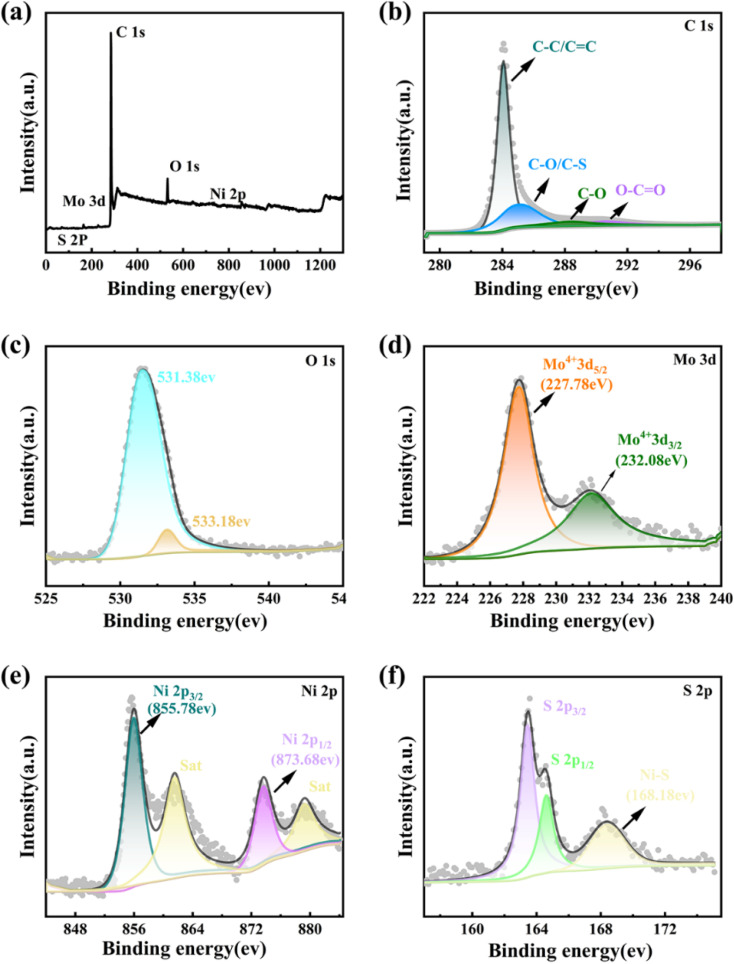
XPS spectra of the MoS_2_ QDs-CNTs/S@NH sample: (a) overall XPS spectrum, (b) C 1s, (c) O 1s, (d) Mo 3d, (e) Ni 2p, and (f) S 2p XPS spectra.

X-ray photoelectron spectroscopy (XPS) was employed to further investigate the chemical composition and surface electric states of the elements in the MoS_2_ QDs-CNTs/S@NH composite. In the overall survey spectrum of Fig. 4(a), the presence of C, O, S, Mo and Ni identifies the existence of these elements in the composite. Fig. 4(b)–(f) display the high-resolution XPS spectra for C 1s, O 1s, Mo 3d, Ni 2p and S 2p of MoS_2_ QDs-CNTs/S@NH, respectively. As shown in Fig. 4(b), the C 1s spectrum can be deconvoluted into four peaks at 284.7, 285.59, 286.28 and 287.88 eV, which corresponded to the C–C/C=C, C–O/C–S, C–O and O–C=O bonds. The existence of S–C brings out the strong interactions between sulfur and the carbon matrix.^51^ In Fig. 4(c), the peak located at the binding energy of 531.38 eV in the O 1s spectrum can be ascribed to the formation of O–H bonds in Ni(OH)_2_. Fig. 4(d) displays the binding energies of Mo 3d_5/2_ and Mo 3d_3/2_ in the material at 227.78 eV and 232.08 eV, respectively. In Fig. 4(e), two prominent peaks of the Ni 2p XPS spectrum labeled as Ni 2p_3/2_ and Ni 2p_1/2_ are located at binding energies of 855.78 eV and 873.68 eV, and the satellite peaks are located at 861.48 eV and 879.38 eV. A change in the chemical valence of Ni indicates that polar Ni(OH)_2_ can serve as a medium or catalyst to transform polysulfides, which is beneficial for accelerating the reaction kinetics and enhancing the electrochemical performance. Fig. 4(f) displays the peaks of S 2p (corresponding to S 2p_3/2_ and S 2p_1/2_) located at 163.48 eV and 164.58 eV. The S 2p energy spectrum exhibits a high-energy peak at 168.48 eV, which can give credit to the formation of Ni–S chemical bonds, proving the highly efficient suppression of active sulfur by MoS_2_ QDs-CNTs/S@NH.

**Fig. 5:**
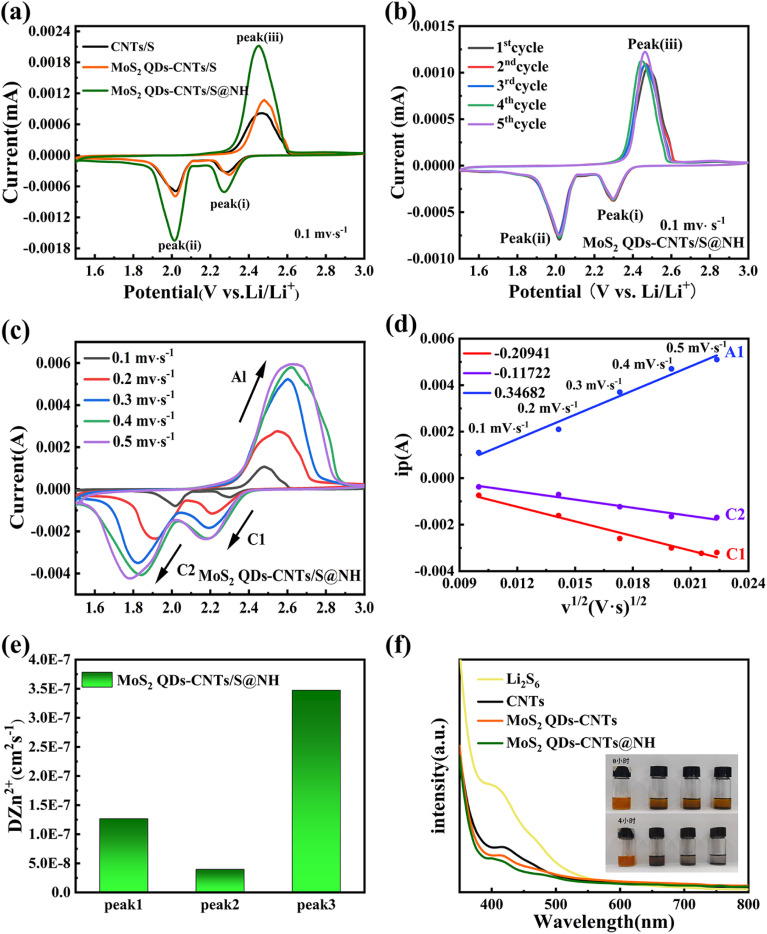
(a) CV curves of CNTs/S, MoS_2_ QDs-CNTs/S, and MoS_2_ QDs-CNTs/S@NH cathodes in the potential range of 1.5–3.0 V at 0.1 mV s^−1^; (b) CV curves at 0.1 mV s^−1^ for MoS_2_ QDs-CNTs/S@NH; (c) CV profiles at different scan rates of MoS_2_ QDs-CNTs/S@NH. (d) Plots of CV peak current *vs.* the square root of the scan rates for MoS_2_ QDs-CNTs/S@NH. (e) Impedance value of MoS_2_ QDs-CNTs/S@NH. (f) UV-vis spectra and variation in the color of Li_2_S_6_ solution adsorbed by CNTs/S, MoS_2_ QDs-CNTs/S, and MoS_2_ QDs-CNTs/S@NH for 4 h.

In order to investigate the electrochemical improvements of the Li–S cells using CNTs/S, MoS_2_ QDs-CNTs/S and MoS_2_ QDs-CNTs/S@NH cathode, cyclic voltammetry (CV) tests were performed at a scan rate of 0.1 mV s^−1^. Fig. 5(a) illustrates the CV curves of cells using CNTs/S, MoS_2_ QDs-CNTs/S and MoS_2_ QDs-CNTs/S@NH as positive electrode materials and lithium foil as the negative electrode. For the three samples, one dominant anodic peak at around 2.45 V corresponds to the oxidation of Li_2_S to S_8_, and two reduction peaks were observed at around 2.29 V and 1.9 V during the discharge process, which correspond to the conversion of elemental sulfur (S_8_) into long-chain Li_2_S_*x*_ (4 < *x* < 8), followed by the further reduction of long-chain lithium polysulfides into short-chain lithium polysulfides, and ultimately to insoluble Li_2_S_2_ and Li_2_S.^52^ Comparatively, the reduction peak of MoS_2_ QDs-CNTs/S@NH shows a higher potential and the oxidation peak shows a lower potential, exhibiting the smallest potential difference between the reduction and oxidation peaks, which suggests the MoS_2_ QDs-CNTs/S@NH has a greater effect on promoting the kinetic reduction of long-chain LiPSs to Li_2_S_2_/Li_2_S and reducing the polarization of the Li–S battery. Meanwhile, the sharpness and intensity of the peaks are significantly higher, indicating faster LiPSs reaction kinetics and smaller electrochemical polarization (Δ*V* = 0.44 for MoS_2_ QDs-CNTs/S@NH; MoS_2_ QDs-CNTs/S Δ*V* = 0.448; CNTs/S Δ*V* = 0.526). These results are consistent with the previous results of Li_2_S_6_ adsorption experiments. Fig. 5(b) illustrates the cyclic voltammetry (CV) curves of MoS_2_ QDs-CNTs/S@NH for the first five cycles in the potential range of 1.5–3 V and a scanning rate of 0.1 mV s^−1^. It can be observed that the first five CV curves overlap well, and the oxidation/reduction peaks exhibit minimal changes, indicating the electrochemical reaction stability during the oxidation–reduction process.

To further investigate the importance of the acceleration of MoS_2_ QDs-CNTs/S@NH on Li^+^ diffusion, CV tests at different scan rates from 0.1 to 0.5 mV s^−1^ were performed. Fig. 5(c) and (d) presents the CV curves of MoS_2_ QDs-CNTs/S@NH at different scanning rates and the corresponding *I*–*v*^1/2^ fitting lines at each redox current peak. In Fig. 5(c), both the positive and negative electrode currents exhibit a more pronounced increasing trend for MoS_2_ QDs-CNTs/S@NH while the scan rate increases, proving the excellent catalytic activity of the MoS_2_ QDs-CNTs/S@NH structure due to the polar and catalytic MoS_2_ QDs decorated on CNTs and a thin Ni(OH)_2_ layer. As shown in Fig. 5(e), the peak current density of MoS_2_ QDs-CNTs/S@NH shows a good linear relationship with the square root of the scanning rate, illustrating that the reaction is a Li^+^ diffusion-controlled process. According to the Randles–Sevcik equation, the diffusion coefficient of lithium-ion (*D*_Li^+^_) could be calculated.^53^ The slopes of *I*–*v*^1/2^ of MoS_2_ QDs-CNTs/S@NH are 0.34682, −0.20941, −0.11722 for peaks A1, C1, C2, suggesting that the MoS_2_ QDs-CNTs/S@NH cathode has a much faster Li^+^ transport.

The CV results verify that the LiPSs have a high chance of being captured using Mo_2_S QDs-CNTs/S@NH as the sulfur host and high-efficiency catalyst. The Mo_2_S QD decorated CNTs network structure with a large surface area provides abundant surface active sites to achieve strong interaction with LiPSs. Meanwhile, Mo_2_S QDs take advantage of their full catalytic activity, which promotes the electron transfer of CNTs networks and then speeds up the conversion from LiPSs to insoluble Li_2_S. Moreover, a thin Ni(OH)_2_ layer coats as a physical barrier, not only restricting sulfur loss but also capturing LiPSs through strong chemical interactions. In addition, the Tafel plots of Mo_2_S QDs-CNTs/S@NH are also tested to demonstrate the enhanced conversion kinetics and electrocatalytic activity (Fig. S2†). The MoS_2_ QDs-CNTs/S@NH displays a much higher *i*_0_ than that of CNTs. The highest exchange current density is −3.647 mA cm^−2^ for the MoS_2_ QDs-CNTs/S@NH electrode. Hence, it turns out that the QDs-CNTs/S@NH can effectively accelerate the reversible electrochemical conversion toward LiPSs.

To further showcase this strong interaction between MoS_2_ QDs-CNTs/S@NHs and polysulfides, a visualized adsorption experiment in Li_2_S_6_ solution (0.01 M) was carried out. Fig. 5(f) displays the photograph of the electrolytes from the three types of samples. The electrolyte using CNTs and MoS_2_ QDs-CNTs cathode exhibited a pale-yellow color. In contrast, the electrolytes used with the MoS_2_ QDs-CNTs@NH cathode remain colorless within 2 h, which indicates that the Li_2_S_6_ in the electrolyte is almost absorbed by MoS_2_ QDs-CNTs@NH. The comparative experiments show that MoS_2_ QDs-CNTs@NH exhibits stronger adsorption capacity and good consistency than CNTs and MoS_2_-CNTs. This is because the MoS_2_ QDs-CNTs@NH can suppress LiPSs dissolution during charge/discharge cycling. As shown in Fig. 5(f), the pure Li_2_S_6_ solution has a significant adsorption peak at 400 nm, which can be attributed to the S_6_^2−^ species. The adsorption strength of UV-vis in the solution with MoS_2_ QDs-CNTs@NH was significantly reduced, which confirmed that the chemisorption capacity was greatly enhanced by MoS_2_ QDs and Ni(OH)_2_ coating.^49^

**Fig. 6:**
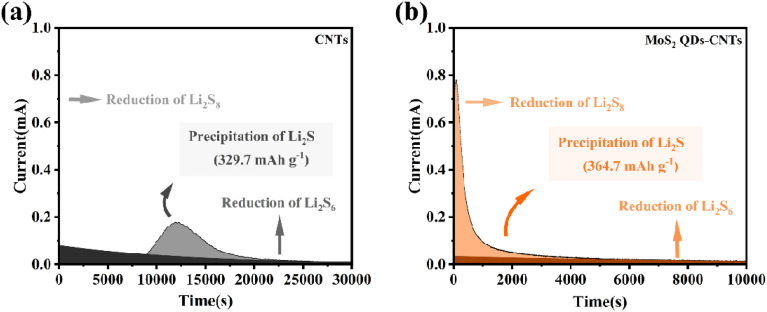
Potentiostatic discharge curves of CNTs and MoS_2_ QDs-CNTs.

In order to further investigate the solid–liquid conversion and Li_2_S deposition kinetics of polysulfides at the electrolyte/electrode interface, the constant potential discharge curves of CNTs and MoS_2_ QDs CNTs surfaces were measured using Li_2_S_6_ as the active substance. As shown in the current time curve in Fig. 6, the dark areas near the *X*-axis and *Y*-axis represent the liquid–liquid reduction reactions of Li_2_S_6_ and Li_2_S_8_, respectively, while the light areas represent the liquid–solid conversion reactions of Li_2_S deposition. In Fig. 6(b), it is worth noting that the MoS_2_ QDs-CNTs have a higher Li_2_S deposition (364.7 mA h g^−1^) than that of CNTs (329.7 mA h g^−1^) (Fig. 6(a)). The results indicated that MoS_2_ QDs were beneficial in achieving efficient Li_2_S precipitation. The oxidative deposition of Li_2_S was further investigated by using a potentiostat, and the dissolved capacity of MoS_2_ QDs-CNTs was much higher than that of CNTs during charging at 2.05 V, revealing the effective oxidation of Li_2_S on the surface of MoS_2_ QDs-CNTs. The catalytic effect of MoS_2_ QDs was demonstrated by the results of the potentiostatic discharge curves of CNTs and MoS_2_ QDs-CNTs.

**Fig. 7:**
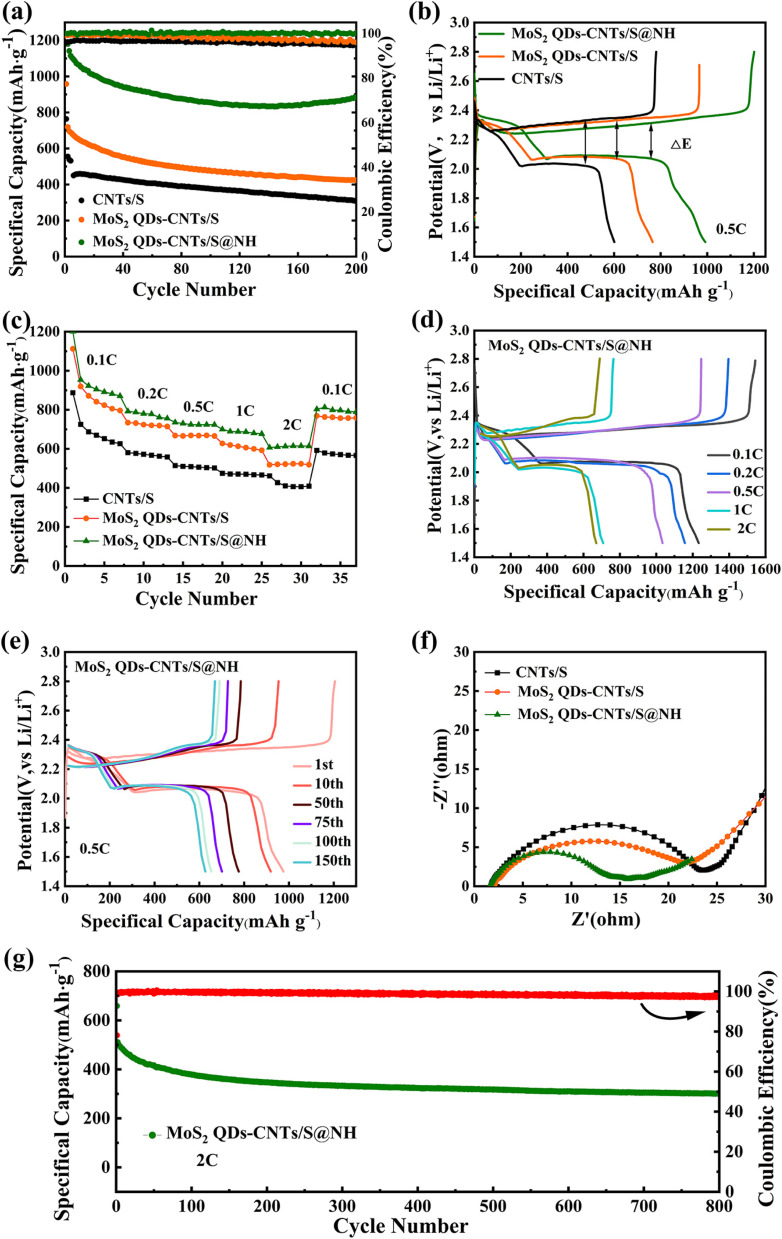
The electrochemical performance of CNTs/S, MoS_2_ QDs-CNTs/S and MoS_2_ QDs-CNTs/S@NH. (a) Cycling performance and coulombic efficiency at 0.5C during 200 cycles, (b) initial discharging/charging curves, and (c) rate capabilities of CNTs/S, MoS_2_ QDs-CNTs/S and MoS_2_ QDs-CNTs/S@NH. (d) Galvanostatic charge–discharge profiles at different rates. (e) Galvanostatic charge–discharge profiles at different cycles of MoS_2_ QDs-CNTs/S@NH. (f) Nyquist plots of CNTs/S, MoS_2_ QDs-CNTs/S and MoS_2_ QDs-CNTs/S@NH electrodes. (g) The long-term cycling performance of the MoS_2_ QDs-CNTs/S@NH cathode at 2.0C.

The cyclic performance of CNTs/S, MoS_2_ QDs-CNTs/S and MoS_2_ QDs-CNTs/S@NH at 0.5C is shown in Fig. 7(a). For MoS_2_ QDs-CNTs/S@NH, the initial discharge capacity of 1141.4 mA h g^−1^ and reversible capacity of 884.6 mA h g^−1^ are obtained after 200 cycles with 77.5% retention. In comparison, the initial discharge capacities of MoS_2_ QDs-CNTs/S and CNTs/S are 719.5 and 555.6 mA h g^−1^. After 200 cycles, their values decrease to 424.1 and 309.8 mA h g^−1^ (retention rates of 58.9% and 55.7%). The poor cycling stability of CNTs/S is attributed to the lack of an effective barrier for polysulfides and relatively slow sulfur redox reactions. Here, the improved cycling stability of MoS_2_ QDs-CNTs/S can be attributed to the designed structure. The CNTs network not only offers rapid electron/ion pathways but also buffers volume changes. The significant number of unsaturated bonds from MoS_2_ QDs efficiently anchor polysulfides, while the external Ni(OH)_2_ shell not only physically confines the active material but also acts as a barrier layer to suppress the diffusion of polysulfides. In addition, the coulombic efficiency of MoS_2_ QDs-CNTs/S@NH during cycling is approximately 99%, which is higher than that of MoS_2_ QDs-CNTs/S and CNTs/S.

Fig. 7(b) presents the initial galvanostatic discharge/charge curves of CNTs/S, MoS_2_ QDs-CNTs/S and MoS_2_ QDs-CNTs/S@NH at 0.2C. Clearly, MoS_2_ QDs-CNTs/S@NH exhibits the longest discharge/charge plateau among the three materials, while MoS_2_ QDs-CNTs/S displays a moderate but longer discharge/charge plateau compared to CNTs/S. These curves exhibit similar shapes at different current densities, clearly showing two distinct discharge voltage plateaus and one charge voltage plateau. In the discharge curves, the two plateaus at approximately 2.31 V and 2.04 V correspond to the two-stage reactions of sulfur to long-chain polysulfides and then to short-chain sulfides. In the charge–discharge plateaus, the two sloping charge plateaus correspond to the oxidation of Li_2_S/Li_2_S_2_ to L_2_S_8_/S, consistent with the analysis from the CV curves. Additionally, MoS_2_ QDs-CNTs/S@NH demonstrates a higher reduction peak potential and a lower oxidation peak potential (Δ*E* = 247.8 mV). As a result, the electrochemical kinetics in MoS_2_ QDs-CNTs/S@NH are greatly enhanced, and the polarization is significantly reduced. Due to the minimal polarization, during the extended discharge plateau, sulfur can be completely converted to insoluble Li_2_S_2_/Li_2_S, thus improving the utilization of active materials and achieving high specific capacity.

Fig. 7(c) displays the rate performance of CNTs/S, MoS_2_ QDs-CNTs/S and MoS_2_ QDs-CNTs/S@NH cathode at different currents. For MoS_2_ QDs-CNTs/S@NH in Fig. 7(d), the reversible discharge/charge capacities provided by the MoS_2_ QDs-CNTs/S@NH cathode are 1061.1/1539.2, 893.7/1201.5, 768.1/1053.9, 697.9/103 and 614.4/941.1 at 0.1, 0.2, 0.5, 1.0 and 2.0C, respectively. When the current density returns to 0.1C, the discharge capacity of the MoS_2_ QDs-CNTs/S@NH recovers to 797.4 mA h g^−1^, demonstrating good reversible capacity. In contrast, both MoS_2_ QDs-CNTs/S and CNTs/S exhibit significantly lower capacities at the same current rate. Clearly, MoS_2_ QDs-CNTs/S@NH displays the best rate performance. The superior rate performance of MoS_2_ QDs-CNTs/S@NH can be primarily attributed to the dual suppression effect, and the dissolution and diffusion of polysulfides are dually suppressed by the internal MoS_2_ QDs and the external Ni(OH)_2_ shell.

For more information about the kinetics of charge transfer and ion diffusion of CNTs/S, MoS_2_ QDs-CNTs/S, and MoS_2_ QDs-CNTs/S@NH cathodes, electrochemical impedance spectroscopy (EIS) was performed. The corresponding results are displayed in Fig. 7(f). The intersection of the plots on the real axis represents the equivalent series resistance (*R*_s_), which includes the electrolyte resistance, the contact resistance and the resistance of the electrode. All three EIS spectra consist of a single semicircle in the high-frequency region and an inclined line in the low-frequency region. The high-frequency semicircle represents the charge transfer resistance (*R*_ct_), and the diagonal line in the low-frequency region is attributed to the Warburg impedance caused by Li^+^ diffusion in the electrode. Part of the irreversible Li_2_S caused the capacity loss, so the *R*_s_ can represent the effectiveness of the redox reaction in the cathode. Clearly, CNTs/S exhibits a relatively high *R*_s_ value of 24 Ω, while MoS_2_ QDs-CNTs/S and MoS_2_ QDs-CNTs/S@NH demonstrate significantly lower *R*_s_ values. Thus, the charge transfer resistance (*R*_ct_) of MoS_2_ QDs-CNTs/S@NH (16 Ω) is lower than that of MoS_2_ QDs-CNTs/S (21 Ω) and CNTs/S (24 Ω). The minimum *R*_ct_ implies the highest electronic conductivity and ionic migration rate between the SEI and electrolyte. These results indicate that constructing a unique core–shell structure of MoS_2_ QDs-CNTs/S@NH can accelerate charge transfer kinetics and inhibit the diffusion of polysulfides.

The long cycling performance of the MoS_2_ QDs-CNTs/S@NH cathode at high current densities was also investigated. As shown in Fig. 7(g), the MoS_2_ QDs-CNTs/S@NH cathode exhibits a capacity of 511.2 mA h g^−1^ and a reversible capacity of 302.2 mA h g^−1^ after 800 cycles at 2C. On average, it retains a capacity retention rate of 59.2%, and a negligible capacity decay of 0.051% per cycle. In addition, the average coulombic efficiency is higher than 97.6%, proving that the shuttling effect has been efficiently suppressed. The results indicate that the remarkable cycling stability of MoS_2_ QDs-CNTs/S@NH at high current densities benefit from the well-designed structures assembled by the number of MoS_2_ QD decorated on CNTs interwoven networks with ultrathin Ni(OH)_2_ coating. This structure can efficiently capture polysulfides through synergistic adsorption and catalysis, which ensures high electrode conductivity, accommodates volume changes, and suppresses shuttle effects. Nanosizing MoS_2_ is the most feasible method to increase its specific surface area and the number of active sites. Due to the fact that the active sites in MoS_2_ are mainly located at the edge sites, reasonable control of its morphology and size is necessary to obtain MoS_2_ nanomaterials rich in edge sites and maximize the exposure of active sites. Many studies have also demonstrated that MoS_2_ nanostructures exhibit good binding strength with polysulfides. As an activated catalyst, it not only promotes the redox kinetics of polysulfides but also facilitates the effective decomposition of lithium sulfide.

## 4 Conclusion

In summary, a new nanocomposite of MoS_2_ QDs-CNTs/S@NH was ingeniously designed as both a sulfur host and catalyst to enhance electrochemical kinetics and trapped LiPSs for Li–S batteries. Combining the advantage of polar and catalytic MoS_2_ QDs with the highly conductive CNTs network, the LiPSs would probably be adsorbed by a large number of unsaturated bonds of MoS_2_ QDs and physical adsorption of conductive CNTs. The thin Ni(OH)_2_ coating forms a protective layer, which not only prevents sulfur loss but also effectively captures LiPSs through chemical interactions. The Li–S battery containing MoS_2_ QDs-CNTs/S@NH as cathode shows outstanding long-term cycling stability and good rate performance. During 800 cycles at 2C, a capacity decay rate of 0.051% per cycle was obtained. This work provides a reasonable design of a sulfur host and shows its potential for application in Li–S batteries.

## Data availability

Data and research materials will be available on request. The data supporting this article have been included in the article and its ESI.†

## Author contributions

Meng Wei: conceptualization, writing – review & editing; Hanqing Lu: investigation; Zhen Wang: formal analysis; Baowen Lu, Pengtao Wang: resources; Xinxin Zhang, Bingjie Feng, Yingjie Xie: validation, Tao Zhang, Guanghui Liu, Song Xu: methodology.

## Conflicts of interest

The authors declare no conflict of interest.

## Supplementary Material

NA-OLF-D4NA00068D-s001
