# Supplementary material for: MoS2 quantum dot-decorated CNT networks as a sulfur host for enhanced electrochemical kinetics in advanced lithium–sulfur batteries
Source: Nanoscale Adv. 2024 Oct 22;6(24):6386–97. doi: 10.1039/d4na00068d (PMC11533053; doi:10.1039/d4na00068d)
Supplement: NA-006-D4NA00068D-s001 [file NA-006-D4NA00068D-s001.pdf]

Supplementary Information (SI) for Nanoscale Advances.
This journal is © The Royal Society of Chemistry 2024

Supporting Information

MoS_2 Quantum Dots-Decorated CNTs Networks as Sulfur Host Enhanced Electrochemical Kinetics for Advanced Lithium–Sulfur Batteries

Meng Wei[1, 2, *], Hanqing Lu[1], Zhen Wang[1], Baowen Lu[1], Pengtao Wang[1], Xinxin Zhang[1],

Bingjie Feng[1], Yingjie Xie[1], Tao Zhang[1], Guanghui Liu[1], Song Xu[1, *]

[1]School of Materials Science and Engineering, Zhengzhou University of Aeronautics, Zhengzhou 450046; Henan Key laboratory of Aeronautical materials and Application Technology, Zhengzhou 450015, China

[2]Collaborative Innovation Center of Aviation Economy Development, Henan Province

[*]Corresponding author: weimeng1005@zua.edu.cn; songxu@zua.edu.cn

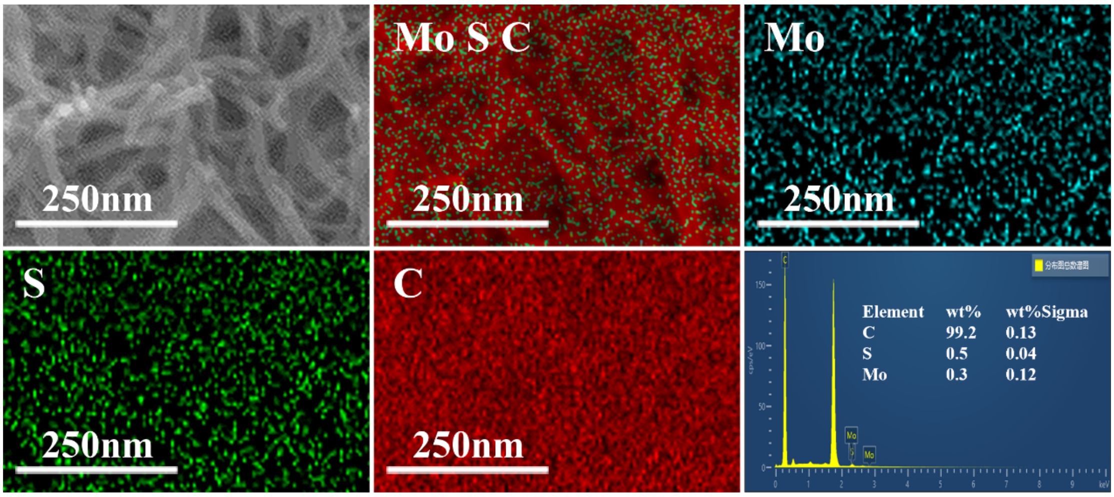

Figure S1 SEM image and corresponding EDS mappings of MoS_2 QDs-CNTs/S.

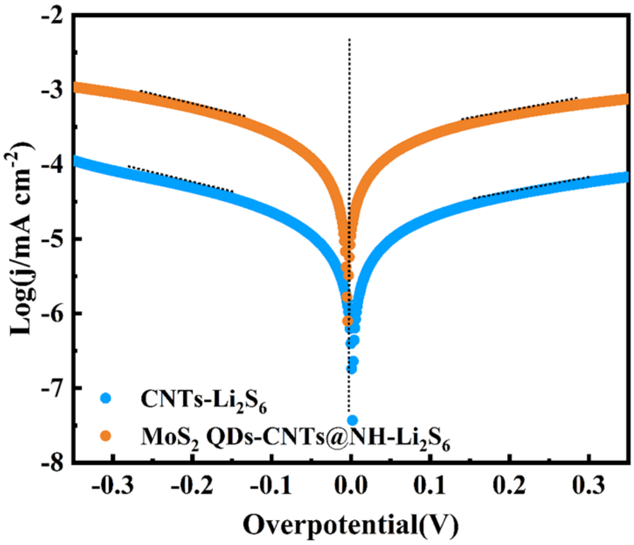

Figure S2 Tafel plots of the Li_2S_6 electrolyte on CNTs and MoS_2 QDs-CNTs@NH surfaces

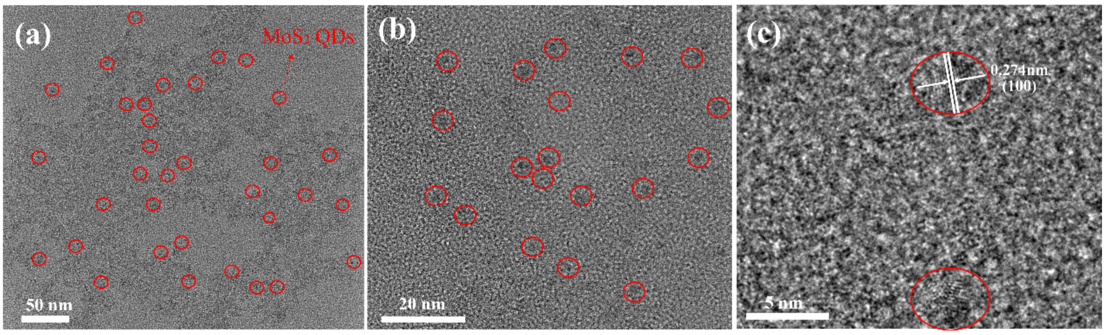

Figure S3 TEM plots of different multiples of MoS_2 QDs

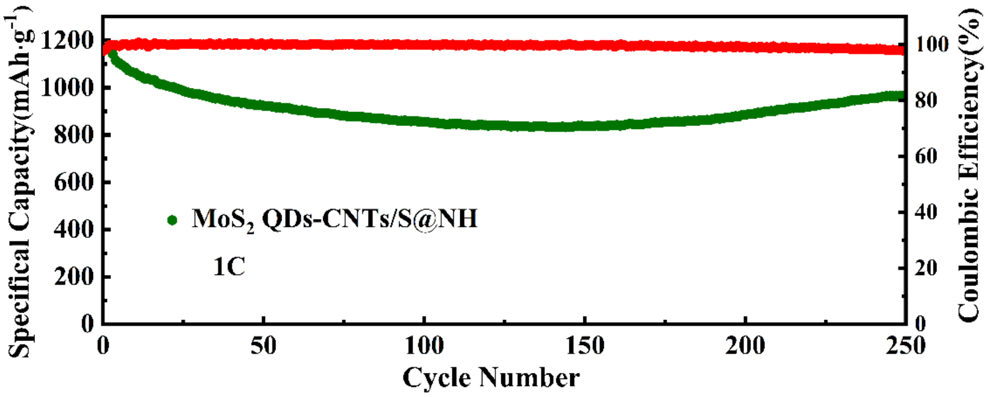

Figure S4 The cycling performance of MoS2 QDs-CNTs/S@NH cathode at 1 C.

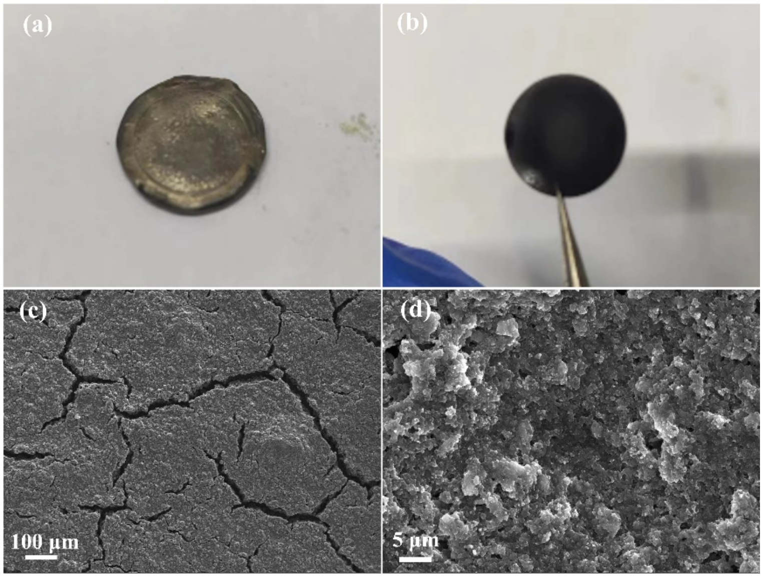

Figure S**5**. morphology of lithium anode and MoS2-CNTs/S@NH composites after 200 cycles at 2 C current density.

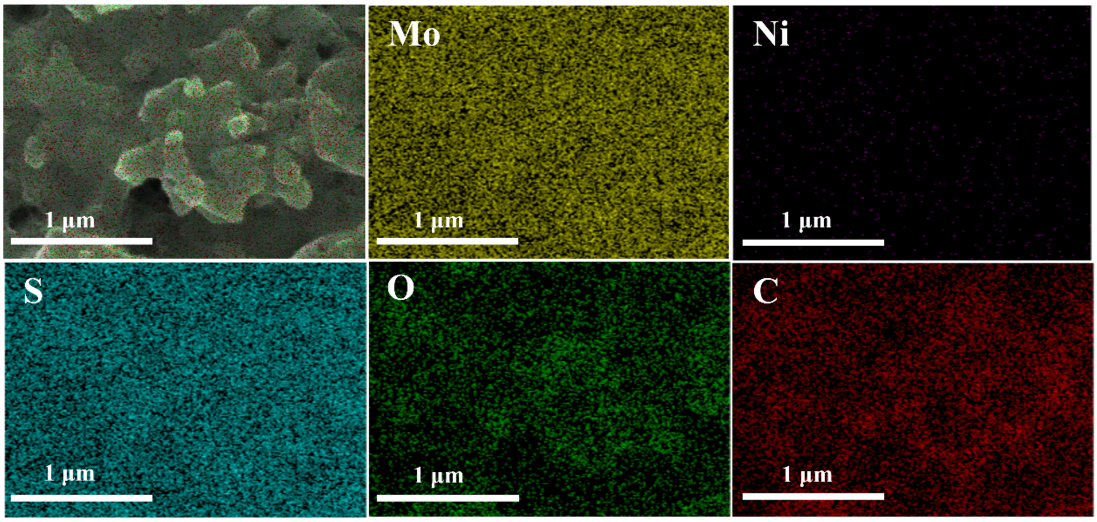

Figure S**6**. The Surface morphology of MoS2 QD-CNTs/S@NH in the discharged state after 200 cycles at 2 C, and its EDS mapping results (Fig. 3f).

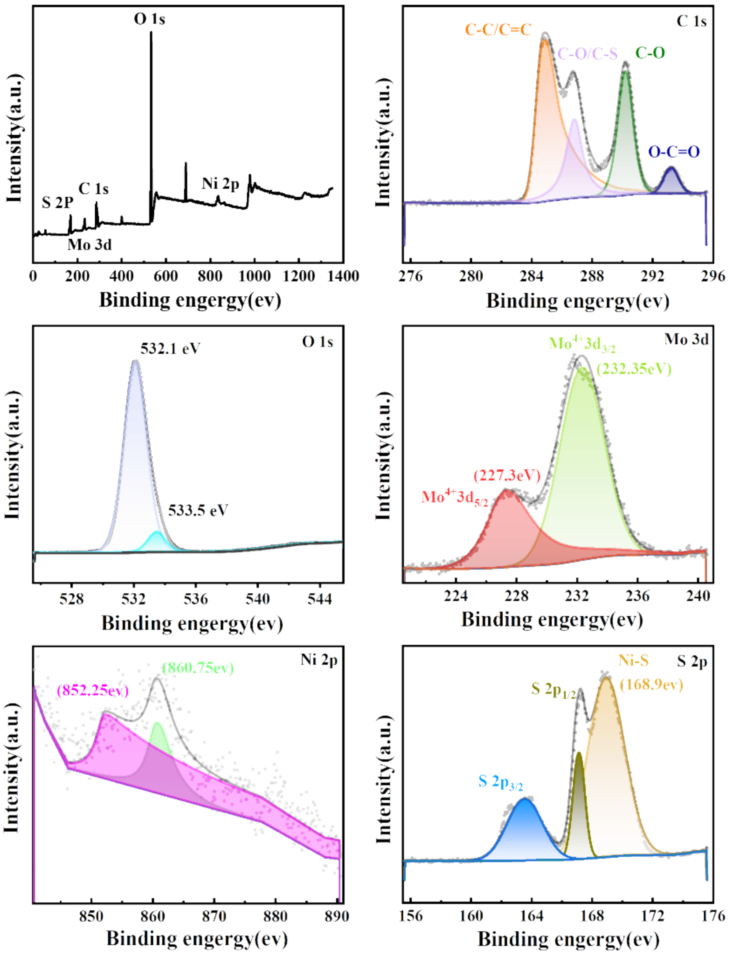

Figure S7. the XPS spectra of the MoS2 QDs-CNTs/S@NH sample after 200 cycles at 2 C current density: (a) overall XPS spectrum, (b) C 1s, © O 1s, (d) Mo 3d, (e) Ni 2p, and (f) S 2p XPS spectra.

TableR1 Electrochemical properties of sulfur-based cathode in this work and literature.

Cathode material	Initial specific capacity /Current density	Capacity after cycling (Cycle number)	Reference
MoS_2 QDs-CNTs/S@$Ni(OH)_2$	1141.4/0.5 C	884.6 (200)	This work
MoS_2/CNTs-S	1271/0.5 C	676.1 (73)	R1
S/MoS_2@G-PCNFs	820/1 C	594 (500)	R2
HFSMS/S	776/0.5 C	430 (200)	R3
CoP@MoS_2-0.75	1016.7/0.5 C	759 (250)	R4
S/CN/MoS_2	930/0.5 C	420 (100)	R5
1T-MoS_2-CNF	890/0.1 C	820 (100)	R6
MoS@rGO/S	1039.1/0.5 C	848.4 (200)	R7
